# Three‐dimensional versus two‐dimensional laparoscopy: What is the evidence?

**DOI:** 10.1111/vsu.14329

**Published:** 2025-08-06

**Authors:** Eric Monnet

**Affiliations:** ^1^ Department of Clinical Sciences, College of Veterinary Medicine Colorado State University Fort Collins Colorado USA

## Abstract

**Background:**

Minimally invasive surgery has made tremendous progress in the last two decades thanks to the more sophisticated instrumentation, new entry ports, vessel sealant devices, and ultrasound dissectors. Cameras and monitors are now high‐definition or 4 K, increasing the clarity of the image available to the surgeon. Those progresses have made minimally invasive surgery safer. However, the biggest challenge of minimally invasive surgery is the lack of depth perception, which requires the surgeon's brain to recreate a three‐dimensional (3D) image using cues in the field. The development of 3D cameras enable surgeons to have a more realistic depth perception. The development of the third generation of 3D cameras, combined with light‐polarizing glasses, resolved the problem of dizziness experienced by surgeons during long and complicated procedures.

**Aims:**

To review the evidence from human and veterinary surgery regarding the benefits of 3D visualization during laparoscopy.

**Conclusions and implications:**

The evidence suggests that 3D visualization enhances depth perception, safety, a smoother learning curve, and overall wellness for surgeons. Some controversies persist on the clinical benefit of 3D visualization, even if most of the studies showed statistically significant reduction of operating time, estimated blood loss, operating errors, and shorten hospital stay in human patients.

Laparoscopy requires the introduction of an endoscope connected to a camera in the abdominal cavity after creation of pneumoperitoneum. The image is projected onto a flat‐screen monitor. Two‐dimensional (2D) cameras have been used for many years. They result in a monocular view of the surgical field, which results in a lack of depth perception. It requires the brain to adapt and recreate a three‐dimensional (3D) view using environmental cues such as cast shadows, occlusion, texture, relative size, distance to the horizon, linear perspective, and motion parallax.^1^, ^2^ A lot of improvement in equipment has been made to facilitate minimally invasive surgery, including more instrumentation, different access ports, vessel sealant devices, and ultrasonic dissectors. High‐definition and 4 K cameras and monitors have been developed to improve the accuracy of images, reducing stress during surgery and also minimizing technical errors during procedures.^3^, ^4^ However, the persistence of monocular visualization continued to affect the depth perception. The lack of stereoscopic visualization makes laparoscopic surgery difficult to learn and stressful.^5^

According to a review from Biospace Olympus Corporation, Karl Storz SE & Co, Richard Wolf GmbH, BBraun Melsungen AG, Stryker, Visionsense, Fujifilm Holdings Corp, Microport, Arthrex Inc., Conmed Corporate, and Sometech Inc. are the key players in the field of 3D laparoscopy. The market of 3D laparoscopy will reach 6.5 billion $ in 2030. (https://www.biospace.com/3d‐laparoscopy‐imaging‐systems‐market‐size‐growth‐trends‐report‐2022‐2030).

A 3D view requires two eyes separated by a 6 cm distance (stereopsis) to provide two different images of the same field to the brain (Figure 1).^6^ The development of three cameras with a binocular visualization helped restore depth perception. A dual‐channel optical scopes use two cameras mounted in the camera handpiece to recreate the binocular vision like the human vision with two eyes (Figure 2). The central unit polarizes the images captured by the 3D camera before being projected on a 3D monitor (Figures 3 and 4). The visualization of 3D images require the use of polarizing glasses by surgeons and assistants, which allows the diffusion of one image to one eye and the other image to the other eye. The brain can then recreate a 3D image and reduce stress during surgery.^7^ The surgeon's subjective assessment of image quality, depth perception, operative strain, ease of intracorporeal suturing, and knotting, and hand‐eye coordination is considered good in most of the cases.^8^ However, for this technology to work properly, it requires good alignment of the surgeons and the assistants with the monitor. If one assistant is not in good alignment with the 3D monitor, the image will not be 3D and will be distorted. The utilization of an extra 2D monitor has been commonly used to assist during the procedure.

**FIGURE 1:**
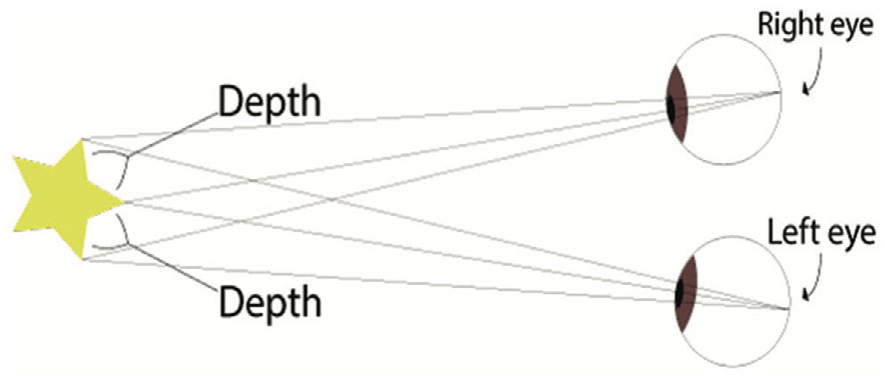
Stereoscopic vision: The perception of depth and three‐dimensional structure is obtained on the basis of visual information derived from both eyes by processing the binocular disparities in the visual cortex (courtesy of Sinha et al.^6^).

**FIGURE 2:**
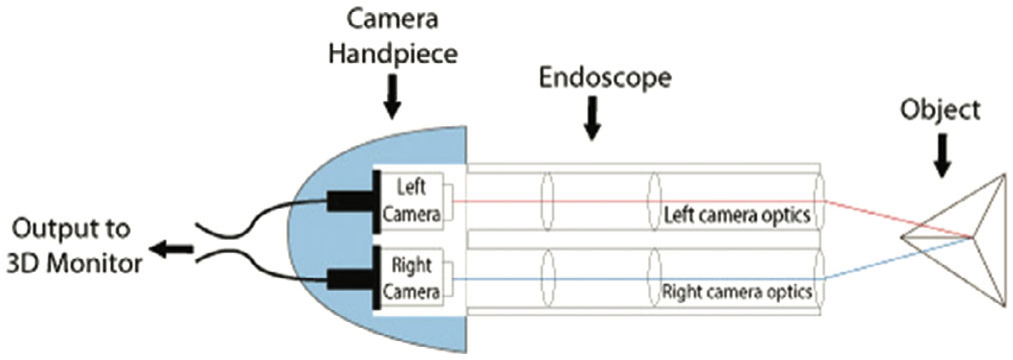
Physics of a three‐dimensional telescope: The telescope has two optical channels that carry two separate images which are fused together to produce a single binocular vision with depth perception (courtesy of Sinha et al.^6^).

**FIGURE 3:**
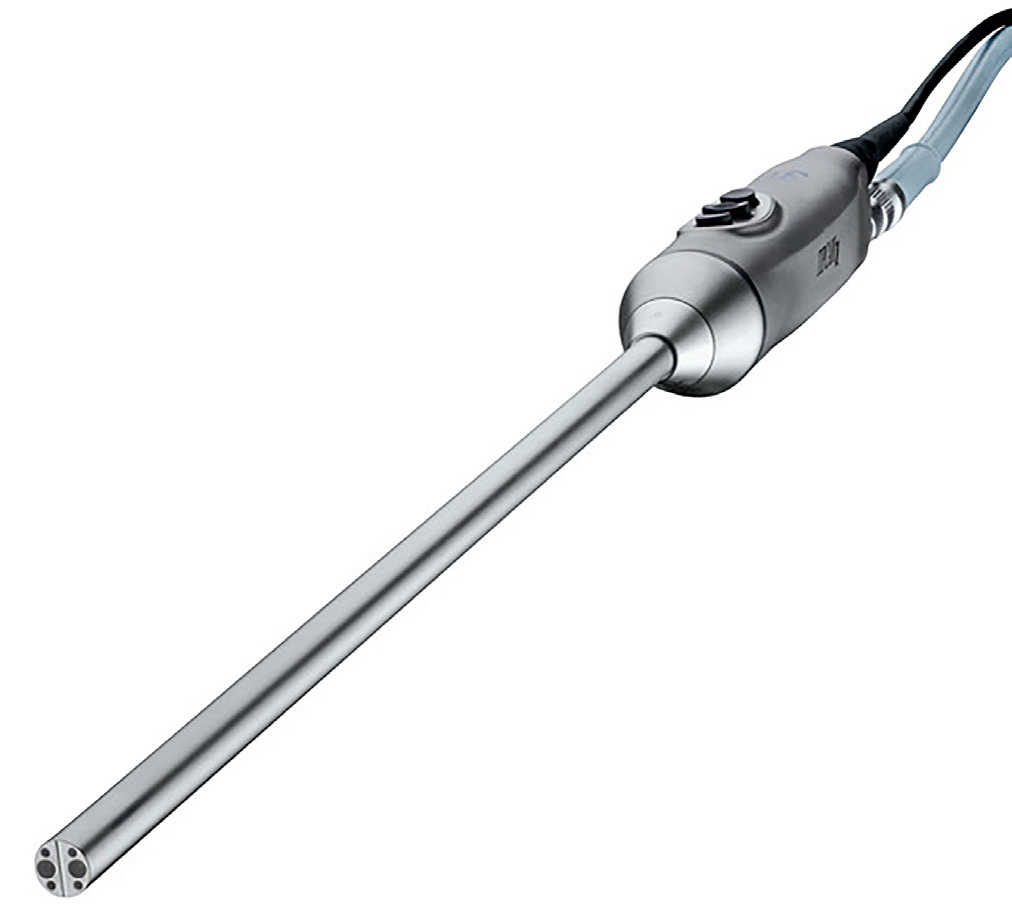
A three‐dimensional telescope and camera with the two optical channels and light sources (courtesy of Karl Storz Vet Endoscopy).

**FIGURE 4:**
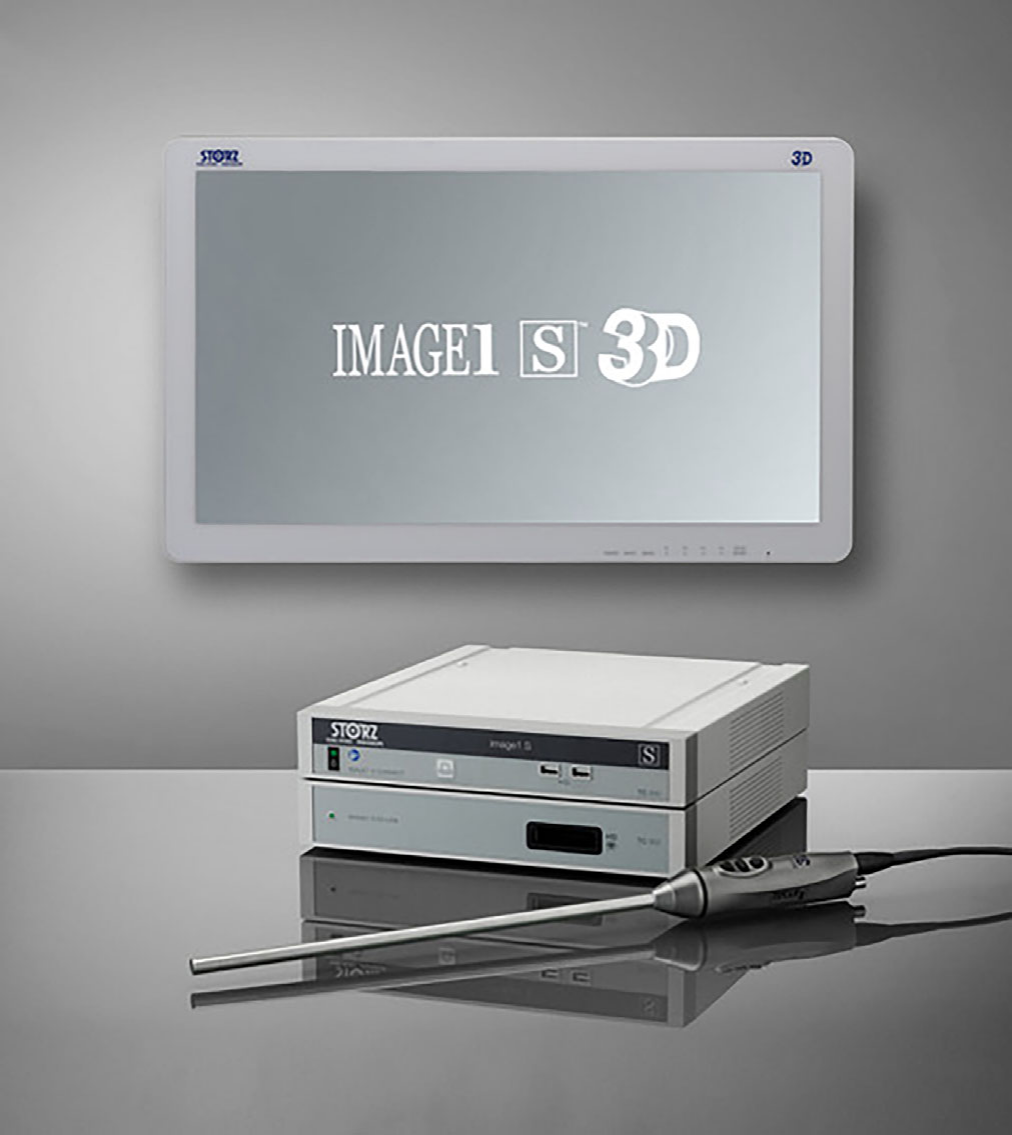
A three‐dimensional (3D) telescope and camera with a control unit and a 3D monitor (courtesy of Karl Storz Vet Endoscopy).

The technology that enables 3D visualization has made significant progress over the last 20 years, making it easier to use and inducing less dizziness in surgeons. Initially, in the first generation of 3D visualization, battery‐operated glasses were used.^2^ In the second generation, helmet‐style head‐mounted displays were used.^2^ Those systems were heavy, not easy to use, and induced a lot of stress to the surgeon, headaches, and motion sickness. The third‐generation system used now only requires polarized light glasses for both the surgeon and the assistant (Figure 5).^2^ A fourth generation is now available with still light polarizing glass but with higher definition.^9^ Additionally, 3D visualization can be combined with near‐infrared light to acquire fluorescent images during minimally invasive surgery (Figure 6).^10^

**FIGURE 5:**
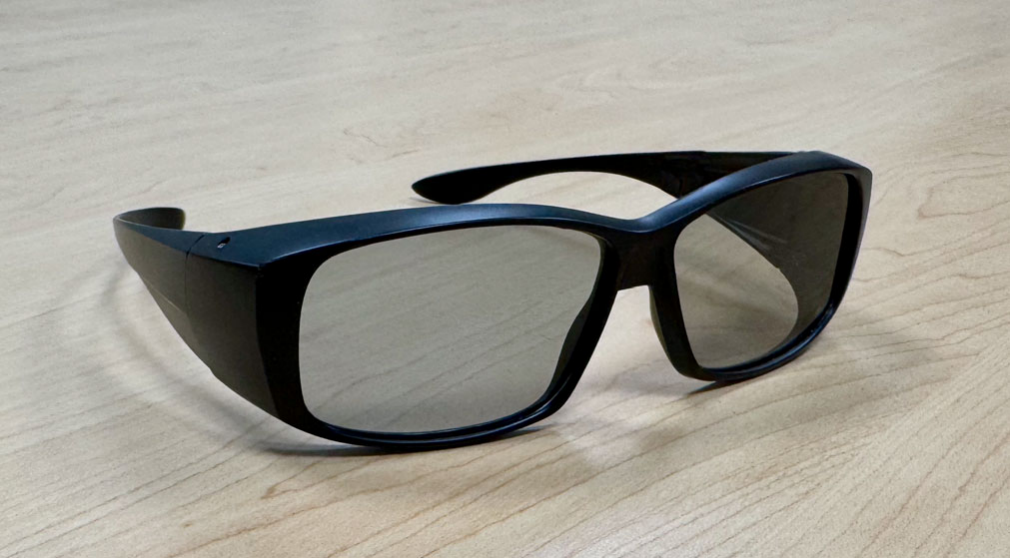
Polarizing glasses used with the third generation of three‐dimensional laparoscopy.

**FIGURE 6:**
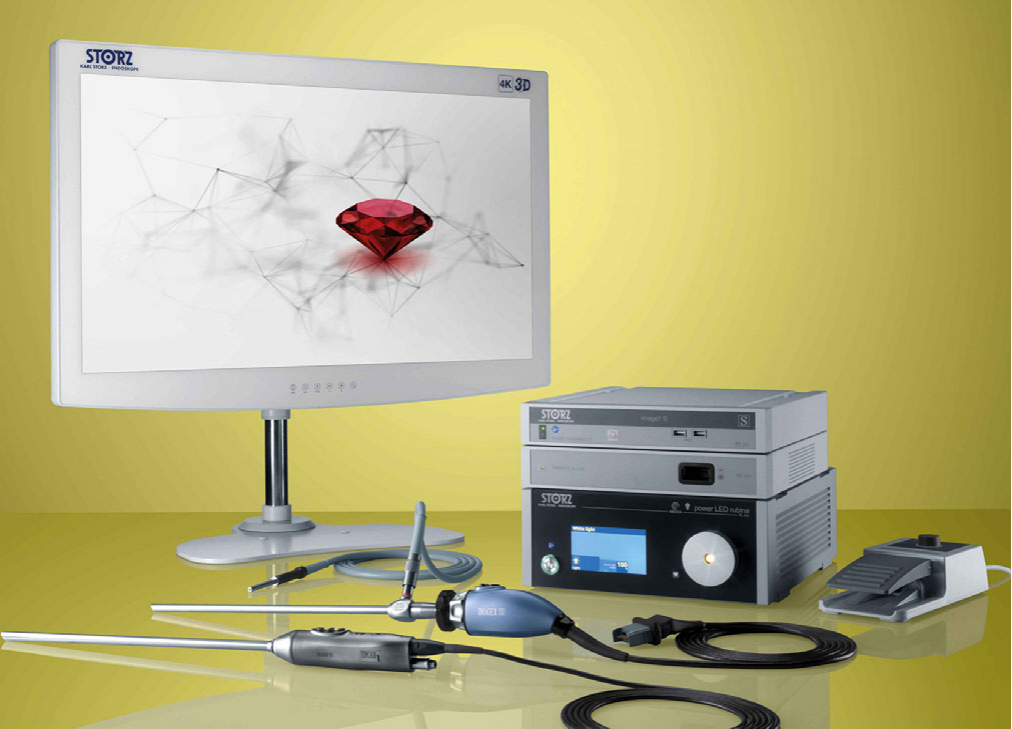
A three‐dimensional (3D) telescope and camera with a control unit, near‐infrared light, and a 3D monitor used for overlay fluorescent imaging (courtesy of Karl Storz Vet Endoscopy).

The benefit of 3D visualization has been compounded in human surgery by the increased utilization of robotic surgery since surgical robots are equipped with 3D cameras. Surgical robots facilitate complex tasks, particularly suturing in challenging locations, which is also aided by 3D visualization.^6^, ^11^, ^12^ A PubMed search including “three‐dimensional” and “laparoscopy” revealed 514 publications in the last 5 years, witnessing how active the field of research about the applications and the benefits of 3D visualization in laparoscopy is. For this review article, we reviewed 25 systematic and or meta‐analyses published over the last decade.^13^, ^14^, ^15^, ^16^, ^17^, ^18^, ^19^, ^20^, ^21^, ^22^, ^23^, ^24^, ^25^, ^26^, ^27^, ^28^, ^29^, ^30^, ^31^, ^32^, ^33^, ^34^, ^35^, ^36^, ^37^ We excluded six meta‐analyses that compared 3D laparoscopy to robotic surgery. Additionally, 41 randomized clinical trials have been published in the last 5 years on a wide variety of surgical techniques, not all of which are particularly relevant to veterinary surgery. Not all randomized clinical trials were included in this study. We entered five studies that were comparing 3D and 2D cameras in an experimental setting with different tasks.^7^, ^31^, ^34^, ^38^, ^39^

Three‐dimensional visualization has been used in a wide variety of laparoscopic procedures. Hepatectomy, bariatric surgery, hernia repair, adrenalectomy, cholecystectomy, nephrectomy, prostatectomy, and colorectal cancer have been the main indications for the utilization of 3D laparoscopy.^29^, ^32^, ^35^, ^40^, ^41^, ^42^, ^43^ It has been shown to be also helpful during single‐site laparoscopy.^39^, ^44^

## 1 EXPERIMENTAL TRIAL ON TASK PERFORMANCE

Three‐dimensional laparoscopy has been associated with some benefits when used in a simulator to perform specific tasks.^7^, ^31^, ^34^, ^38^, ^39^, ^45^ In most of the studies, the level of training was documented. Usually, the tasks performed were described by the Fundamentals of Laparoscopic Surgery (FLS) (https://www.sages.org/wiki/fundamentals-laparoscopic-surgery/). It commonly included the peg transfer, pattern cutting, endoloop placement, and intracorporeal suturing. Usta et al.^38^ used 10 tasks (FLS tasks plus bead transfer, needle placement, passing of needle into loops, and catching a dropped needle) in their study to compare the effects of 3D cameras, and they used eight participants with lot of experience, eight with moderate of experience, and eight without experience.

The time to complete each task was measured, and the number of errors was documented. Additionally, various criteria, often subjective, were used to assess the depth perception by the surgeon performing the tasks. All studies in simulators demonstrated a reduction in time to complete tasks with 3D visualization.^7^, ^31^, ^34^, ^38^, ^39^ The suturing time was mainly improved.^7^, ^31^ It also reduced the number of errors in completing each task. ^7^, ^31^, ^34^, ^38^, ^39^ Vilaca et al.^36^ also reported that 3D visualization improves comfort for the surgeon. All the studies concluded that 3D visualization was beneficial for the learning curve, even if it was beneficial for expert surgeons. Depth perception was the main subjective advantage of the 3D visualization.^7^, ^31^, ^34^, ^38^, ^39^ Usta et al.^38^ reported that depending on the level of difficulty of the task, 3D visualization helped the less experienced participants with the simpler tasks and the most experienced surgeons with the most difficult procedures. However, Restaino et al.^34^ concluded in their meta‐analysis that the benefits observed in simulators do not always transfer to hysterectomy and vaginal cuff suturing in patients.

## 2 CLINICAL TRIALS ON OPERATIVE OUTCOMES

### 2.1 Review of meta‐analysis

The meta‐analysis reviewed for this article included studies on bariatric surgery, cholecystectomy, liver lobectomy, colectomy, colorectal surgery, nephrectomy, hysterectomy, and prostatectomy in human patients.^11^, ^12^, ^13^, ^14^, ^15^, ^16^, ^17^, ^18^, ^19^, ^20^, ^21^, ^22^, ^23^, ^24^, ^25^, ^26^, ^27^, ^28^, ^29^, ^30^, ^31^, ^32^, ^33^, ^34^ Those meta‐analyses have reviewed several prospective randomized clinical trials to extract mainly the surgical time, the amount of blood loss, and the complications. Most meta‐analyses demonstrated a statistically significant reduction in operative time. For cholecystectomy, it has been shown that 3D visualization significantly reduces surgical time by 4.23 min and also reduces the time to expose the Calot's triangle by 4.19 min.^22^ It also significantly lowers the risk of gallbladder perforation with a risk ratio of 0.50. In their review, Patel et al.^31^ showed minimal improvement in operating time with 3D visualization for cholecystectomy, with great variability in the number of errors happening during surgery. This variability was mostly due to the criteria used to evaluate the errors. For radical prostatectomy, the use of 3D visualization reduced operative time by 42.85 min and estimated blood loss by 78.38 mL. Three‐month postoperative continence recovery was significantly higher with 3D visualization than with 2D visualization.^17^ In the same analysis, operating time and estimated blood loss were not affected by the 3D visualization of pyeloplasty and partial nephrectomy.^17^ Cheng et al.^11^ reviewed 21 prospective, randomized clinical trials to evaluate the efficacy of 3D visualization for a wide variety of abdominal surgeries. Operative time, estimated blood loss, the number of postoperative complications, and the length of hospital stay were significantly reduced with the use of 3D visualization. The operative time was significantly reduced from 2.8 min for urological procedures to 14.1 min for digestive surgeries.^11^ Estimated blood loss was significantly reduced by 7.05 mL for digestive surgery to 103.62 mL for prostatectomy.^11^ However, it was increased by 24 mL for other urological surgeries.^11^ However, the amount of abdominal drainage, the duration of drainage, the number of lymph nodes removed, and the anastomotic time for prostatectomy were not affected by the type of visualization used.^11^ In a meta‐analysis on liver lobectomy, Chan et al.^32^ showed that operating time, estimated blood loss, and need for reoperation were not improved with 3D visualization; however, overall morbidity was reduced. In an analysis on bariatric surgery, the operating time was reduced from 12 to 36 min, depending on the study, with a standardized mean difference of 1.19 in favor of the 3D visualization.^33^ It also resulted in shorter hospital stays. In a meta‐analysis on colorectal surgery, six studies that included 614 patients were retained, showing that 3D visualization resulted in a minimal reduction in operating time from 8 to 20 min, similar estimated blood loss, but an increased number of lymph nodes resected when compared to 2D visualization.^26^ The rate of conversion to an open surgery, the length of hospital stay, and the rate of postoperative complications were not affected by the utilization of 3D laparoscopy.^26^

### 2.2 Review of prospective randomized clinical trials

Several studies in human surgery have looked at the benefits of 3D visualization in different prospective randomized studies.^6^, ^11^, ^29^, ^35^, ^40^, ^41^, ^42^, ^43^, ^46^, ^47^, ^48^, ^49^, ^50^ It has been shown to improve the accuracy and safety of the procedure. This has been documented by evaluating complication rates intra‐ and postoperatively, the time of surgery, and the estimated amount of blood loss during the procedure.^6^, ^11^, ^29^, ^35^, ^40^, ^41^, ^42^, ^43^, ^46^, ^47^, ^48^, ^49^, ^50^ In a study on colonic surgery, 3D visualization reduced the operating time from 142 to 123 min but it had no effect on the estimated blood loss, the number of postoperative complications, and the number of lymph nodes resected.^9^ Ajao et al.^51^ showed in a randomized clinical trial on hysterectomy that 3D visualization did not seem to help the surgeons in training to complete the suturing of the vaginal cuff. Obviously, there are some controversies between studies about the benefit of 3D versus 2D visualization.^17^ Those discrepancies can come from several reasons, but most of all it is coming from a lack of standardization to evaluate the benefit of 3D visualization.

### 2.3 Surgeon's perspective

Most of the retrospective randomized clinical trials tried to document subjectively the surgeon's perspective. Most of the surgeons reported a better depth perception, less eye fatigue, and less dizziness with 3D visualization. Objective data is lacking to truly document the impact on the surgeon's wellness. The evaluation of the benefit of 3D visualization is compounded by many factors, including the stereoacuity of each individual. Apparently, 30% of the population have reduced stereoacuity, and 3% are stereoblind.^2^, ^52^, ^53^ “Stereoacuity refers to the smallest disparity an individual can detect. Under optimal conditions, a trained observer can reliably resolve a disparity between 2 and 6 arc sec. A disparity of 2 arc sec corresponds to the depth interval of 4 mm viewed from 5 m away.”^53^

## 3 EXPERIENCE IN VETERINARY SURGERY

Three‐dimensional laparoscopy has been previously reported in veterinary surgery.^54^, ^55^, ^56^ When used for intracorporeal gastropexy, 3D laparoscopy did not result in a shorter surgical time.^54^ However, the workload measured with the NASA task load index was significantly reduced by 3D visualization.^54^ In a study on cholecystectomy in canine cadavers, 3D visualization only reduced the surgical time from the introduction of the instruments in the abdominal cavity to the placement of the first clip on the cystic duct; the total surgical time was not affected.^56^ During radical prostatectomy in canine cadavers, the utilization of 3D laparoscopy improved the safety of the procedure because it allowed for a more accurate placement of the suture during the vesico‐urethral anastomosis, reducing the risk of iatrogenic injury to the ureters.^55^

In conclusion, 3D visualization improves depth perception, maintains tactile feedback, improves safety, and enhances the wellness of the surgeon. There is no clear evidence that 3D visualization improves the clinical outcome of the procedures. Some statistically significant differences reported in several studies may not translate into significant clinical improvement.

## AUTHOR CONTRIBUTIONS

The author has reviewed all the literature cited in this review and wrote the manuscript.

## CONFLICT OF INTEREST STATEMENT

The author declares no conflict of interest.

## References

[1] Bogdanova R , Boulanger P , Zheng B . Depth perception of surgeons in minimally invasive surgery. Surg Innov. 2016;23:515‐524. 27009686 10.1177/1553350616639141

[2] Sakata S , Watson MO , Grove PM , Stevenson ARL . The conflicting evidence of three‐dimensional displays in laparoscopy: a review of systems old and new. Ann Surg. 2016;263:234‐239. 26501704 10.1097/SLA.0000000000001504

[3] van Bergen P , Kunert W , Buess GF . The effect of high‐definition imaging on surgical task efficiency in minimally invasive surgery: an experimental comparison between three‐dimensional imaging and direct vision through a stereoscopic TEM rectoscope. Surg Endosc. 2000;14:71‐74. 10653241 10.1007/s004649900015

[4] Usta TA , Gundogdu EC . The role of three‐dimensional high‐definition laparoscopic surgery for gynaecology. Curr Opin Obstet Gynecol. 2015;27:297‐301. 26107783 10.1097/GCO.0000000000000189

[5] Feng X , Morandi A , Boehne M , et al. 3‐dimensional (3D) laparoscopy improves operating time in small spaces without impact on hemodynamics and psychomental stress parameters of the surgeon. Surg Endosc. 2015;29:1231‐1239. 25673344 10.1007/s00464-015-4083-3

[6] Sinha RY , Raje SR , Rao GA . Three‐dimensional laparoscopy: principles and practice. J Minim Access Surg. 2017;13:165‐169. 27143695 10.4103/0972-9941.181761 PMC5485803

[7] Cologne KG , Zehetner J , Liwanag L , Cash C , Senagore AJ , Lipham JC . Three‐dimensional laparoscopy: does improved visualization decrease the learning curve among trainees in advanced procedures? Surg Laparosc Endosc Percutan Tech. 2015;25:321‐323. 26053113 10.1097/SLE.0000000000000168

[8] Nguyen DH , Nguyen BH , van Nong H , et al. Three‐dimensional laparoscopy in urology: initial experience after 100 cases. Asian J Surg. 2019;42:303‐306. 29807690 10.1016/j.asjsur.2018.04.012

[9] Wang Z , Liang J , Chen J , Mei S , Liu Q . Three‐dimensional (3D) laparoscopy versus two‐dimensional (2D) laparoscopy: a single‐surgeon prospective randomized comparative study. Asian Pac J Cancer Prev. 2020;21:2883‐2887. 33112544 10.31557/APJCP.2020.21.10.2883 PMC7798154

[10] Esposito C , Alberti D , Settimi A , et al. Indocyanine green (ICG) fluorescent cholangiography during laparoscopic cholecystectomy using RUBINA™ technology: preliminary experience in two pediatric surgery centers. Surg Endosc. 2021;35:6366‐6373. 34231069 10.1007/s00464-021-08596-7 PMC8523512

[11] Diaz‐Arrastia C , Jurnalov C , Gomez G , Townsend C . Laparoscopic hysterectomy using a computer‐enhanced surgical robot. Surg Endosc. 2002;16:1271‐1273. 12085153 10.1007/s00464-002-8523-5

[12] Sinha A , West A , Vasdev N , et al. Current practises and the future of robotic surgical training. Surgeon. 2023;21:314‐322. 36932015 10.1016/j.surge.2023.02.006

[13] Cheng J , Gao J , Shuai X , Wang G , Tao K . Two‐dimensional versus three‐dimensional laparoscopy in surgical efficacy: a systematic review and meta‐analysis. Oncotarget. 2016;7:70979‐70990. 27486967 10.18632/oncotarget.10916 PMC5342603

[14] Kramp KH , van Det MJ , Hoff C , Veeger NJGM , ten Cate Hoedemaker HO , Pierie JPEN . The predictive value of aptitude assessment in laparoscopic surgery: a meta‐analysis. Med Educ. 2016;50:409‐427. 26995481 10.1111/medu.12945

[15] Sorensen SM , Savran MM , Konge L , et al. Three‐dimensional versus two‐dimensional vision in laparoscopy: a systematic review. Surg Endosc. 2016;30:11‐23. 25840896 10.1007/s00464-015-4189-7

[16] Fergo C , Burcharth J , Pommergaard HC , Kildebro N , Rosenberg J . Three‐dimensional laparoscopy vs 2‐dimensional laparoscopy with high‐definition technology for abdominal surgery: a systematic review. Am J Surg. 2017;213:159‐170. 27816196 10.1016/j.amjsurg.2016.07.030

[17] Yim C , Lo CH , Lau MH , Fan R , Lai HM , Foo DCC . Three‐dimensional laparoscopy: is it as good as it looks?—a review of the literature. Ann Laparosc Endosc Surg. 2017;2:131.

[18] Bertolo R , Checcucci E , Amparore D , et al. Current status of three‐dimensional laparoscopy in urology: an ESUT systematic review and cumulative analysis. J Endourol. 2018;32:1021‐1027. 30064256 10.1089/end.2018.0374

[19] Dirie NI , Wang Q , Wang S . Two‐dimensional versus three‐dimensional laparoscopic Systems in Urology: a systematic review and meta‐analysis. J Endourol. 2018;32:781‐790. 29969912 10.1089/end.2018.0411 PMC6156697

[20] Liang H , Liang W , Lei Z , et al. Three‐dimensional versus two‐dimensional video‐assisted endoscopic surgery: a meta‐analysis of clinical data. World J Surg. 2018;42:3658‐3668. 29946785 10.1007/s00268-018-4681-z

[21] Vettoretto N , Foglia E , Ferrario L , et al. Why laparoscopists may opt for three‐dimensional view: a summary of the full HTA report on 3D versus 2D laparoscopy by S.I.C.E. (Società Italiana di Chirurgia Endoscopica e Nuove Tecnologie). Surg Endosc. 2018;32:2986‐2993. 29368286 10.1007/s00464-017-6006-y PMC5956063

[22] Vettoretto N , Reggiani L , Cirocchi R , et al. Three‐dimensional versus two‐dimensional laparoscopic right colectomy: a systematic review and meta‐analysis. Int J Colorectal Dis. 2018;33:1799‐1801. 29998352 10.1007/s00384-018-3121-8

[23] Chen L , Li B , Zeng L , et al. Three‐dimensional vs 2‐dimensional laparoscopic gastrectomy for gastric cancer: a systematic review and meta‐analysis. Medicine (Baltimore). 2019;98:e18222. 31804348 10.1097/MD.0000000000018222 PMC6919538

[24] Li L , Gao X , Guo Y , et al. Comparison of three‐dimensional versus two‐dimensional laparoscopic surgery for rectal cancer: a meta‐analysis. Int J Colorectal Dis. 2019;34:1577‐1583. 31342167 10.1007/s00384-019-03353-8

[25] Davies S , Ghallab M , Hajibandeh S , Hajibandeh S , Addison S . Three‐dimensional versus two‐dimensional imaging during laparoscopic cholecystectomy: a systematic review and meta‐analysis of randomised controlled trials. Langenbeck's Archives of Surgery/Deutsche Gesellschaft Fur Chirurgie. 2020;405:563‐572. 10.1007/s00423-020-01909-9 32572555

[26] Pantalos G , Patsouras D , Spartalis E , et al. Three‐dimensional versus two‐dimensional laparoscopic surgery for colorectal cancer: systematic review and meta‐analysis. In Vivo. 2020;34:11‐21. 31882458 10.21873/invivo.11740 PMC6984079

[27] Zhao B , Lv W , Mei D , et al. Comparison of short‐term surgical outcome between 3D and 2D laparoscopy surgery for gastrointestinal cancer: a systematic review and meta‐analysis. Langenbeck's Archives of Surgery / Deutsche Gesellschaft Fur Chirurgie. 2020;405:1‐12. 10.1007/s00423-020-01853-8 31970475

[28] Zu G , Jiang K , Zhou T , Che N , Zhang X . Two‐dimensional versus three‐dimensional laparoscopic gastrectomy in surgical efficacy for gastric cancer: a systematic review and meta‐analysis. Clin Transl Oncol. 2020;22:122‐129. 31066012 10.1007/s12094-019-02116-9

[29] Sánchez‐Margallo FM , Durán Rey D , Serrano Pascual Á , Mayol Martínez JA , Sánchez‐Margallo JA . Comparative study of the influence of three‐dimensional versus two‐dimensional urological laparoscopy on surgeons' surgical performance and ergonomics: a systematic review and meta‐analysis. J Endourol. 2021;35:123‐137. 32799686 10.1089/end.2020.0284

[30] Piramide F , Kowalewski KF , Cacciamani G , et al. Three‐dimensional model‐assisted minimally invasive partial nephrectomy: a systematic review with meta‐analysis of comparative studies. Eur Urol Oncol. 2022;5:640‐650. 36216739 10.1016/j.euo.2022.09.003

[31] Singla V , Bhattacharjee HK , Gupta E , Singh D , Mishra AK , Kumar D . Performance of three‐dimensional and ultra‐high‐definition (4K) technology in laparoscopic surgery: a systematic review and meta‐analysis. Journal of Minimal Access Surgery. 2022;18:167‐175. 35313429 10.4103/jmas.jmas_122_21 PMC8973492

[32] Chan KS , Shelat VG . Three‐dimensional versus two‐dimensional laparoscopy in laparoscopic liver resection: a systematic review and meta‐analysis. J Laparoendosc Adv Surg Tech A. 2023;33:678‐690. 37057963 10.1089/lap.2023.0081

[33] Patel M , Hugh TJ . A comparison of three‐dimensional visualization systems and two‐dimensional visualization systems during laparoscopic cholecystectomy: a narrative review. J Laparoendosc Adv Surg Tech A. 2023;33:957‐962. 37486672 10.1089/lap.2023.0270

[34] Restaino S , Scutiero G , Taliento C , et al. Three‐dimensional vision versus two‐dimensional vision on laparoscopic performance of trainee surgeons: a systematic review and meta‐analysis. Updates Surg. 2023;75:455‐470. 36811183 10.1007/s13304-023-01465-z

[35] Peltrini R , Esposito MD , Pacella D , Calabrese P , Vitiello A , Pilone V . Three‐dimensional versus two‐dimensional laparoscopic bariatric surgery: a systematic review and meta‐analysis. Obes Surg. 2024;34:2177‐2185. 38630144 10.1007/s11695-024-07222-4 PMC11127895

[36] Tercan C , Gunes AC , Bastu E , Blockeel C , Aktoz F . The comparison of 2D and 3D systems in total laparoscopic hysterectomy: a systematic review and meta‐analysis. Arch Gynecol Obstet. 2024;310:1811‐1821. 39180564 10.1007/s00404-024-07630-y

[37] Thrikandiyur A , Kourounis G , Tingle S , Thambi P . Robotic versus laparoscopic surgery for colorectal disease: a systematic review, meta‐analysis and meta‐regression of randomised controlled trials. Ann R Coll Surg Engl. 2024;106:658‐671. 38787311 10.1308/rcsann.2024.0038 PMC11528374

[38] Usta TA , Ozkaynak A , Kovalak E , Ergul E , Naki MM , Kaya E . An assessment of the new generation three‐dimensional high definition laparoscopic vision system on surgical skills: a randomized prospective study. Surg Endosc. 2015;29:2305‐2313. 25414065 10.1007/s00464-014-3949-0

[39] Vilaca J , Leite M , Correia‐Pinto J , et al. The influence of 3D in single‐port laparoscopy surgery: an experimental study. Surg Laparosc Endosc Percutan Tech. 2018;28:261‐266. 29782431 10.1097/SLE.0000000000000536

[40] Koppatz HE , Harju JI , Sirén JE , Mentula PJ , Scheinin TM , Sallinen VJ . Three‐dimensional versus two‐dimensional high‐definition laparoscopy in transabdominal preperitoneal inguinal hernia repair: a prospective randomized controlled study. Surg Endosc. 2020;34:4857‐4865. 31754852 10.1007/s00464-019-07266-z PMC7572346

[41] Materazzi G , Rossi L . Robot‐assisted adrenalectomy: state of the art. Updates Surg. 2021;73:1131‐1146. 33175318 10.1007/s13304-020-00915-2 PMC8184704

[42] Eskelinen M , Saimanen I , Selander T , et al. Three‐dimensional laparoscopy (3D‐LC) versus minilaparotomy (MC) in cholecystectomy: a prospective randomized study. In Vivo. 2022;36:2835‐2839. 36309399 10.21873/invivo.13022 PMC9677764

[43] Sapci I , GamalEldin M , Rencuzogullari A , et al. Prospective randomized comparison of three‐dimensional (3D) versus conventional laparoscopy in total colectomy for ulcerative colitis. ANZ J Surg. 2023;93:2155‐2160. 36898957 10.1111/ans.18368

[44] Vilaça J , Pinto JP , Fernandes S , Costa P , Pinto JC , Leão P . Comparative study of 2D and 3D optical imaging systems: Laparoendoscopic single‐site surgery in an ex vivo model. Surg Innov. 2017;24:598‐604. 28871872 10.1177/1553350617728160

[45] Dion YM , Gaillard F . Visual integration of data and basic motor skills under laparoscopy. Influence of 2‐D and 3‐D video‐camera systems. Surg Endosc. 1997;11:995‐1000. 9381356 10.1007/s004649900510

[46] Lusch A , Bucur PL , Menhadji AD , et al. Evaluation of the impact of three‐dimensional vision on laparoscopic performance. J Endourol. 2014;28:261‐266. 24059674 10.1089/end.2013.0344

[47] Baum S , Sillem M , Ney JT , et al. What are the advantages of 3D cameras in gynaecological laparoscopy? Geburtshilfe Frauenheilkd. 2017;77:45‐51. 28190888 10.1055/s-0042-120845 PMC5292906

[48] Chan AC , Chung SC , Yim AP , et al. Comparison of two‐dimensional vs three‐dimensional camera systems in laparoscopic surgery. Surg Endosc. 1997;11:438‐440. 9153170 10.1007/s004649900385

[49] Honeck P , Wendt‐Nordahl G , Rassweiler J , Knoll T . Three‐dimensional laparoscopic imaging improves surgical performance on standardized ex‐vivo laparoscopic tasks. J Endourol. 2012;26:1085‐1088. 22721451 10.1089/end.2011.0670

[50] Lee CL , Desai T , Huang KG . The role of three‐dimensional laparoscopy in gynecology: time to revise our perspective? Gynecol Minim Invasive Ther. 2024;13:1‐3. 38487617 10.4103/gmit.gmit_99_23 PMC10936727

[51] Ajao MO , Larsen CR , Manoucheri E , et al. Two‐dimensional (2D) versus three‐dimensional (3D) laparoscopy for vaginal cuff closure by surgeons‐in‐training: a randomized controlled trial. Surg Endosc. 2020;34:1237‐1243. 31172324 10.1007/s00464-019-06886-9

[52] Zaroff CM , Knutelska M , Frumkes TE . Variation in stereoacuity: normative description, fixation disparity, and the roles of aging and gender. Invest Ophthalmol vis Sci. 2003;44:891‐900. 12556426 10.1167/iovs.02-0361

[53] Grove PM . The psychophysics of binocular vision. In: Zhu C , Zhao Y , Yu L , et al., eds. 3D‐TV System with Depth‐Image‐Based Rendering: Architectures, Techniques and Challenges. Springer New York; 2013:347‐373 New York, NY.

[54] Balsa IM , Giuffrida MA , Mayhew PD . A randomized controlled trial of three‐dimensional versus two‐dimensional imaging system on duration of surgery and mental workload for laparoscopic gastropexies in dogs. Vet Surg. 2021;50:944‐953. 33864647 10.1111/vsu.13637

[55] Monnet E , Hafez A . Description of the technique for laparoscopic radical prostatectomy in canine cadavers: 2D vs. 3D camera. PLoS One. 2022;17:e0274868. 36445893 10.1371/journal.pone.0274868 PMC9707773

[56] Azuma K , Monnet E . Three‐dimensional versus two‐dimensional laparoscopy for cholecystectomy in a canine cadaveric study. Vet Surg. 2024;53:695‐700. 37985468 10.1111/vsu.14046

